# The Utility of Molecular Imaging in Prostate Cancer

**DOI:** 10.1007/s11934-015-0573-z

**Published:** 2016-02-19

**Authors:** Aaron Leiblich, Daniel Stevens, Prasanna Sooriakumaran

**Affiliations:** Department of Urology, Churchill Hospital, University of Oxford NHS Trust, Old Road, Headington, Oxford, UK; Department of Physiology, Anatomy and Genetics, University of Oxford, Le Gros Clark Building, South Parks Road, Oxford, OX1 3QX UK; Nuffield Department of Surgical Sciences, University of Oxford, John Radcliffe Hospital, Oxford, OX3 9DU UK

**Keywords:** Surgery, Prostate cancer, MRI, PET/CT, PSMA

## Abstract

Prostate cancer is the commonest solid-organ cancer diagnosed in males and represents an important source of morbidity and mortality worldwide. Imaging plays a crucial role in diagnosing prostate cancer and informs the ongoing management of the disease at all stages. Several novel molecular imaging technologies have been developed recently that have the potential to revolutionise disease diagnosis and the surveillance of patients living with prostate cancer. These innovations include hyperpolarised MRI, choline PET/CT and PSMA PET/CT. The major utility of choline and PSMA PET/CT currently lies in their sensitivity for detecting early recurrence after radical treatment for prostate cancer and identifying discrete lesions that may be amenable to salvage therapy. Molecular imaging is likely to play a future role in characterising genetic and biochemical signatures in individual tumours, which may be of particular significance as cancer therapies move into an era of precision medicine.

## Introduction

Prostate cancer is the most frequently diagnosed malignancy in men and is the second commonest cause of male cancer-related deaths in Europe and North America [1, 2]. Disease that is organ-confined at the time of diagnosis is associated with a good prognosis and is frequently amenable to curative therapy [2, 3]. Conversely, metastatic disease heralds a poor prognosis; men with skeletal metastases have a 5-year survival rate below 30 % [4, 5].

Molecular imaging describes a discipline that emerged in the early 2000s and facilitates the visualisation of biological interactions at the cellular level [6]. Unlike traditional imaging modalities, such as ultrasound or conventional CT, molecular imaging techniques are able to probe the biochemical abnormalities that underpin a particular disease process. Clearly, this will prove an invaluable tool for planning individualised treatments as we progress toward an era of personalised medicine, whereby cancer is viewed as a genetically and biochemically heterogeneous disease best managed with tailored therapies [7–10].

This review aims to discuss molecular imaging techniques currently employed in the assessment of prostate cancer and to also familiarise the reader with ongoing developments that are likely to have clinical application in the near future.

## Molecular Imaging as a Diagnostic and Staging Tool in Newly Diagnosed Prostate Cancer

Radiological imaging plays an important role in the diagnosis and initial staging of prostate cancer. For many decades, trans-rectal ultrasound (TRUS)-guided biopsy has served as the standard technique for obtaining prostatic tissue for diagnosis. In recent years, multi-parametric MRI (MP-MRI) has emerged as a vital tool for the diagnosis and accurate staging of prostate cancer [11, 12]. To date, no CT- and/or PET-based imaging modalities have demonstrated any benefit over MRI in the imaging of early prostate cancer [13–15]. However, advances in MR spectroscopy seem likely to permit technologies for use in clinical contexts that provide data on tumour metabolism, thus helping to establish the aggressiveness of discrete malignant foci.

A promising development in this area is the emergence of hyperpolarised [1-^13^C] pyruvate as an adjunct to conventional MR. Hyperpolarised MR is a novel molecular imaging technique that can be used to monitor the metabolism of endogenous biomolecules [16•, 17]. Pre-clinical mouse models of prostate cancer have described increased signal detected by MR from hyperpolarised lactate in malignant relative to normal tissues, with levels of both hyperpolarised lactate and pyruvate seen to increase with progression and reduce after therapy [18, 19]. A promising study in humans demonstrates the safety and feasibility of hyperpolarised pyruvate injections in men with prostate cancer [16•]. This study demonstrates that elevated levels of hyperpolarised pyruvate are observed in regions of biopsy-proven prostate cancer. In addition to its use as a diagnostic tool, it seems probable that refinements in this approach will provide insights into individual tumour biology and behaviour. If this comes to fruition, hyperpolarised MR will undoubtedly aid the risk stratification of organ-confined malignancy at the time of diagnosis and also serve as a powerful tool to monitor disease in patients managed on an active surveillance protocol.

## Detecting Early Recurrence After Radical Therapy

The aim of radical treatment in prostate cancer is to cure men of their disease. Unfortunately, a proportion of men will develop disease relapse despite radical therapy. Therefore, an important aspect of the management of men who have undergone radical therapy involves surveillance and detection of recurrence. A rising PSA level after radical therapy is indicative of disease relapse. The management of recurrent disease will depend on several factors, including the PSA velocity and extent and location of disease [20]. Advances in molecular imaging provide new opportunities to assess the extent and distribution of recurrent disease. Choline PET/CT is a technique that is becoming increasingly employed as a standard imaging modality in cases of suspected relapse. More recently, the imaging agent Ga-68 prostate-specific membrane antigen (Ga-68 PSMA) used in conjunction with PET/CT has been shown to detect early recurrence with very high sensitivity [21].

## Choline PET Scanning as a Tool for Identifying Early Recurrence

A study from 2011 evaluated 43 patients who underwent radical prostatectomy with 31 days after C11-choline PET scan [22]. Trans-axial images and histological specimens were analysed by comparing the respective slices. This study demonstrated that small tumours (<5 mm) were poorly detected while larger tumours tended to be well delineated by PET.

The greatest utility of choline PET scanning appears to be in the context of rising PSA following definitive local therapy. This is of particular use in patients with biochemical relapse, whereby PSA elevation is the only manifestation of their disease. In this context, choline PET scanning can help distinguish between localised, regional and distant disease recurrence. This informs disease management as the extent of recurrence helps define treatment options. For example, small areas of local recurrence may be amenable to salvage radiotherapy, whereas early detection of distant disease may facilitate the timely introduction of anti-androgen therapy (ADT) [23]. In one study of 63 patients with biochemical relapse, 56 % (35/63) had abnormal choline PET scans. Recurrent disease could be localised in 36 % of patients with PSA value <1 ng/ml, 43 % with PSA value between 1 and 2 ng/ml, 62 % with PSA value between 2 and 3 ng/ml and 73 % with a PSA value above 3 ng/ml [24].

An additional study of 170 patients with biochemical failure post-radical prostatectomy demonstrated that 44 % of patients had a positive C11-choline PET scan. Interestingly, there appears to be an association between PSA doubling time (PSADT) and the ability of choline PET to detect recurrence. The percentage of patients with positive PET scans in whom the PSADT was >6 months was just 27 %, compared to 81 % where the PSADT was <3 months [25]. Similarly, a correlation exists between PSA velocity (PSAV) and positive choline PET scans in patients with biochemical relapse after prostatectomy—patients with positive C11-choline PET scans have significantly higher PSAV (*p* < 0.05) than patients with negative scans [26]. These data suggest a relationship between PSA kinetics and choline uptake in the context of biochemical relapse. In summary, choline PET scanning is increasingly employed as a method for localising recurrent tumours in patients with biochemical relapse. Already, choline PET is being used to help plan whether with recurrent disease patients would benefit from localised or systemic therapies. A drawback of this technique is that it only appears to be useful once a diagnosis of biochemical relapse has been made and is currently not able to detect recurrent disease before a rise in PSA is noted.

## Prostate-Specific Membrane Antigen PET (PSMA PET)—a Technique for Detecting Very Early Recurrence

Although choline-based PET imaging is increasingly used for the detection of recurrent disease following curative therapy, several studies have reported concerns regarding sensitivity and specificity at low PSA levels [27–29]. Consequently, there is a need for a reliable diagnostic test in patients presenting with early biochemical failure. With this in mind, radionuclide imaging agents that target the cell surface receptor PSMA are receiving increasing attention [30••, 31, 32]. PSMA is expressed in the prostate, salivary glands, small intestine and kidneys. However, it is significantly overexpressed in prostate cancer cells when compared to the surrounding normal tissue [33, 34]. PSMA is a trans-membrane receptor that internalises bound ligands [35]. It is the internalisation of the ligand-radionuclide complex that is thought to account for the high-quality images produced by this technique [36].

Ga-68 PSMA-targeted diagnostic imaging has many advantages over traditional PSA testing, not least the ability to detect anatomically the location of lymph node metastases and distant soft tissue spread. Historically, salvage radiation therapy has been delivered to the prostatic bed without knowledge of the site of residual disease. This new modality opens the possibility of salvage surgical or radiation therapy that can be targeted to the correct location [37]. Two groups have published papers in 2015 examining the effectiveness of Ga-68 PSMA imaging for detecting recurrent prostate cancer [30••, 32]. Firstly, Ashfar-Oromich et al. performed a retrospective analysis in 319 patients who underwent Ga-68 PSMA imaging [30••]. In 292 of these patients, progressive disease was suspected following prior conventional treatment (e.g., radiation therapy/surgery). Ga-68 identified positive lesions in 50 % of men with a PSA <0.2 ng/ml, and as expected, the likelihood of a positive lesion correlated to PSA levels. From this cohort, tissue from 42 patients was isolated for further histological testing (biopsy/surgery) allowing comparison of images with gold standard histology. Lesion-based analysis from these 42 men gave sensitivity, specificity, negative predictive value and positive predictive values of 76, 100, 91 and 100 %, respectively. Secondly, Dietlein et al. have published a comparison of two PSMA-labelled radionuclides (Ga-68 and F-18). In 14 men with biochemical relapse, both PSMA-labelled isotopes identified positive lesions in 10 patients [32]. This study was limited by only two patients having histological confirmation of their positive lesions but did demonstrate the ability of this technology to highlight sites of residual disease or metastasis. Further studies are required with histological verification of positive lesions before it is adopted into standard practice.

## Detecting Skeletal Metastases and Other Distant Disease

As alluded to earlier, the skeleton is a frequent site of metastases in prostate cancer, and the development of bony disease is an indicator of poor prognosis. For many years, conventional wisdom has held that the presence of skeletal spread indicates terminal disease. However, recent interest has emerged in the possibility of identifying patients with a low burden of metastatic disease (so-called oligo-metastases), with the aim of offering potentially curative or disease-controlling therapy [38–42]. Clearly, the success of this philosophy is likely to depend on the ability to detect bone disease as early as possible. The involvement of the skeleton in prostate cancer is an important cause of morbidity and mortality [43]. Skeletal involvement in prostate cancer is typically difficult to assess using traditional imaging modalities, such a plain X-ray and CT, especially in the context of osteoblastic metastases [44]. Scintigraphy has long been a mainstay of assessing bone disease through the use of radiolabelled Tc99m-methylene diphosphate (MDP) [45]. MDP uptake by the skeleton is a function of blood supply, bone turnover and osteoblastic activity. Although this technique is sensitive at detecting osteogenic activity, there are limitations. MDP bone scan findings reflect changes in the bone rather than visualising the tumour. Disease regression can be difficult to discern due to residual tracer uptake in healing bone [46]. It is unlikely that conventional scintigraphy possesses the sensitivity and specificity to detect tiny skeletal deposits of prostate cancer. To this end, molecular imaging modalities offer great promise in the early detection of bone metastases.

## F18-Sodium Fluoride (NaF) PET Scanning for Imaging Skeletal Disease

Interest in F18-sodium fluoride (NaF) PET scanning as a tool for imaging prostate cancer bone metastases has grown in recent years. Like radiolabelled phosphate analogues, NaF is incorporated into the hydroxyapatite lattice and collagen matrix of the skeleton. NaF has a higher affinity to bone than MDP and therefore allows earlier imaging time and improved image quality [47]. Indeed, several studies have indicated that NaF PET performs better than MDP for the assessment of osteoblastic metastases. One notable study compares planar bone scintigraphy, bone SPECT, NaF PET and NaF-PET/CT in patients with localised, high-risk or metastatic prostate cancer [48]. The sensitivity and specificity for detecting bone lesions were significantly higher for NaF PET/CT (100 and 100 %, respectively) than for planar scintigraphy (70 and 57 %), SPECT (92 and 82 %) or NaF PET alone (100 and 62 %). These results favour NaF PET/CT over conventional scintigraphy for the accurate detection of skeletal disease in prostate cancer.

Although NaF PET/CT is generally regarded as superior to conventional NDP scintigraphy, no prospective studies yet exist demonstrating any benefit in patient staging or management and it is unclear whether NaF will provide more meaningful information. Further clarity is needed to demonstrate a definitive advantage of NaF over conventional bone scanning techniques, which are cheaper and more widely available.

## FDG-PET to Monitor Treatment Response in Advanced Disease States

Many prostate tumours possess low glycolytic activity, leading to the widely held notion that FDG-PET scanning has limited value in the assessment of prostate cancer. This is likely to be true in the case of organ-confined disease, where tumours may display low levels of glycolysis and benign hyperplastic or inflammatory pathologies may lead to increased FDG uptake, leading to false-positive diagnoses [49, 50]. However, FDG-PET may play a role in advanced disease states, where it has been used to assess response to hormone and/or chemotherapy in the context of metastatic disease [51].

## The Future—Probing Genetic Abnormalities in Prostate Cancer

Molecular imaging techniques currently used in the clinical setting to assess prostate cancer largely employ the use of substrates taken up by metabolising tissues. This approach risks non-specificity for malignant cells, as well as missing tumours with low metabolic turnover. To this end, molecular probes are being developed that specifically target antigens selectively expressed by prostate cancer cells. In addition, cancer-specific probes are likely to be of great value as a tool to delineate the genetic heterogeneity of individual tumours and thus facilitate targeted, precision therapies [8, 9].

## Androgen Receptor (AR) Probes

AR signalling is frequently implicated as a driver of tumour growth in prostate cancer, and anti-androgen therapy has long been a mainstay of the treatment of advanced disease. Invariably, after a period of response to hormone therapy, prostate tumours progress to a castrate-resistant state. This complex process is thought to involve either sensitisation or bypassing of the AR signalling pathway [52].

The distribution of sites where AR is overexpressed can be imaged using the PET agent [^18^F]-fluoro-5-alpha-dihydrotestosterone (18F-FDHT) [53, 54]. A recent study evaluated the associations between morphological CT patterns, glycolytic activity and AR expression on PET and found that the number of bone lesions on CT, FDG-PET and FDHT-PET as well as the intensity of FDHT uptake are significantly associated with overall survival [55•]. Of particular promise is the potential use of FDHT as a pharmacodynamic marker of drug targeting. One study reported that enzalutamide (an AR receptor antagonist)-induced FDHT uptake changes in metastatic lesions could be considered a surrogate marker for response of prostate cancer metastases with AR overexpression to ADT [56].

## Conclusions

Prostate cancer is an important cause of morbidity and mortality affecting men worldwide. Herein, we have discussed several new developments in molecular imaging techniques that are likely to see increasing adoption over the next decade and that have good potential to revolutionise the management of men with prostate cancer.

In the future, molecular imaging techniques are likely to play a role in the management of prostate cancer at all stages of the disease, from the use of hyperpolarised MR in organ-confined disease, to the use of choline or PSMA tracers to detect early recurrence, right through to the detection of distant disease with NaF and FDG-PET. Additionally, imaging techniques using cancer-specific molecular probes (AR probes are an example) are currently being developed that will provide a non-invasive method for sub-typing tumours based on genetic or biochemical signatures. Such advances will help move us into the era of precision medicine, whereby therapies are tailored according to the specific characteristics of any given tumour.
